# Strenuous running exacerbates knee cartilage erosion induced by low amount of mono-iodoacetate in rats

**DOI:** 10.1186/s12891-017-1393-8

**Published:** 2017-01-25

**Authors:** Ryusuke Saito, Takeshi Muneta, Nobutake Ozeki, Yusuke Nakagawa, Mio Udo, Katsuaki Yanagisawa, Kunikazu Tsuji, Makoto Tomita, Hideyuki Koga, Ichiro Sekiya

**Affiliations:** 1 Department of Joint Surgery and Sports Medicine, Graduate School of Medicine, Tokyo Medical and Dental University, Tokyo, Japan; 2 Center for Stem Cell and Regenerative Medicine, Tokyo Medical and Dental University, 1-5-45 Yushima, Bunkyo-ku, Tokyo, 113-8510 Japan; 3 Department of Cartilage Regeneration, Graduate School of Medicine, Tokyo Medical and Dental University, Tokyo, Japan; 4 Clinical Research Center, Tokyo Medical and Dental University, Tokyo, Japan

**Keywords:** Osteoarthritis, Rat, Cartilage, Strenuous running, Mono-iodoacetate (MIA), Synovitis

## Abstract

**Background:**

It is still debated whether strenuous running in the inflammatory phase produces beneficial or harmful effect in rat knees. We examined (1) the dropout rate of rats during a 30-km running protocol, (2) influences of strenuous running and/or low amounts of mono-iodoacetate injection on cartilage, and (3) the effect of strenuous running on synovitis.

**Methods:**

Rats were forced to run 30 km over 6 weeks and the dropout rate was examined. One week after 0.1 mg mono-iodoacetate was injected into the right knee, rats were forced to run either 15 km or not run at all over 3 weeks, after which knee cartilage was evaluated. Synovium at the infrapatellar fat pad was also examined histologically.

**Results:**

Even though all 12 rats run up to 15 km, only 6 rats completed 30 km of running. Macroscopically, 0.1 mg mono-iodoacetate induced erosion at the tibial cartilage irrespective of 15 km of running. Histologically, 0.1 mg mono-iodoacetate induced loss of cartilage matrix in the tibial cartilage, and an additional 15 km of strenuous running significantly exacerbated the loss. Synovitis caused by mono-iodoacetate improved after running.

**Conclusions:**

Only 50% of rats completed 30 km of running because of foot problems. Strenuous running further exacerbated tibial cartilage erosion but did not influence synovitis induced by mono-iodoacetate.

## Background

Osteoarthritis (OA) is a multifactorial, progressive, and painful disease. OA is influenced by genetic and environmental factors, including mechanical stress [1]. To overcome difficulties in studying osteoarthritis in humans, a variety of animal models have been developed [2–4]. The use of strenuous running helps simulate long-term stress on weight-bearing joints. This model does not require surgical procedures; therefore it can detect subtle symptoms of osteoarthritis without surgical modification. Running protocols for rats usually require 30 km in 6 weeks (Table 1), but a proportion of rats often drop off the protocol because of foot problems, and quantitative data for this dropout rate have not been reported [5, 6]. We previously used 30 km of running protocol and experienced drop out problems. Here, we first examined the dropout rate of rats during a forced 30-km running protocol.

**Table 1:** Reports on the effects of running exercise on articular cartilage in Wistar rats

Study	Running distance	Running period	OA induced	Method	Effect
Sekiya et al. [5] 2009	30 km	6 weeks	No		Harmful
Siebelt et al. [24] 2011	31.8 km	6 weeks	No		Harmful
Beckett et al. [17] 2012	30 km / 55 km	3 / 6 weeks	No		Harmful
Pap et al. [20] 1998	30 km	12 weeks	Yes	Intracranial self stimulation	Harmful
Siebelt et al. [21] 2014	15 km	6 weeks	Yes	Papain injection	Harmful
Saito et al. 2015	15 km	3 weeks	Yes	MIA 0.1 mg injection	Harmful
Galois et al. [22] 2004	15 km	4 weeks	Yes	ACLT	Beneficial
Cifuentes et al. [23] 2010	15 km	8 weeks	Yes	MIA 1.2 mg injection	Beneficial

*Abbreviations*: *ACLT* anterior cruciate ligament transection, *MIA* mono-iodoacetate

OA progression is also influenced by inflammation, which is possibly affected by mechanical stress [7]. Acute inflammation is one of the triggers for OA. Intra-articular injection of mono-iodoacetate (MIA) induces arthritis and 1 mg MIA is often used to induce arthritis in rats. However, in this condition, both cartilage and bone are rapidly destructed [8–10]. Therefore, the use of this amount seems to be inappropriate for analyzing OA progression in OA models. It is still debated whether strenuous running in the inflammatory phase produces beneficial or harmful effect in rat knees (Table 1). We hypothesized that it is possible to reduce the running distance required to make OA in combination with another OA inducer as MIA. If both strenuous running and low amounts of MIA induce OA in an additive manner, this will be a good model for mimicking OA in rats. Secondly, we examined the influences of strenuous running and/or low amounts of MIA injection on cartilage.

It is widely accepted that synovial inflammation is a feature of OA [11–13]. Clinically, exercise is effective in improving symptoms of OA in the chronic phase [14, 15]. On the other hand, it is well known that exercise in the inflammatory phase leads to exacerbation of symptoms [16]. However, it is still unknown how exercise affects synovitis. Thirdly, we examined if low amounts of MIA injection induced synovitis and how strenuous running affected synovitis, if at all.

## Methods

### Animals

All animal care and experiments were conducted in accordance with the institutional guidelines of the Animal Committee of Tokyo Medical and Dental University. Thirty-six wild type male Wistar rats (Charles River Laboratories Japan, Kanagawa, Japan) from the ages of ten to eleven weeks were used for the experiments. The weight ranged between 294-342 g. Rats were housed under a 12-h light–dark cycle and allowed food and water ad libitum.

### 30 km running of rats

**Fig. 1:**
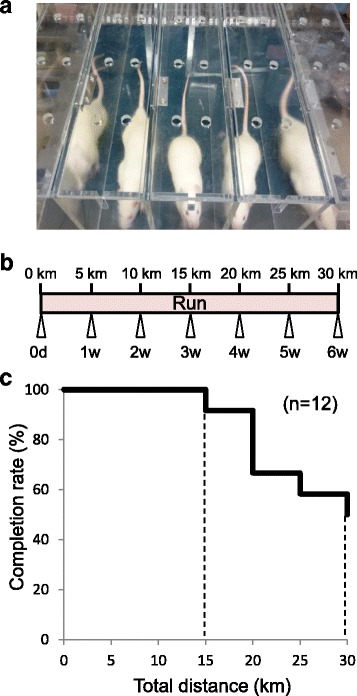
Relationship between the number of rats that could continue to run and the distance rats run during strenuous running. **a** Treadmill for rats. **b** Protocol of strenuous running for 30 km for 6 weeks. **c** Survival rate of rats during the strenuous running

For strenuous running, we used a rodent treadmill machine (MK-680R5; ME Service, Tokyo, Japan), in which electrical shocks were applied to the grid behind the lane to stimulate the rats that failed to run spontaneously (Fig. 1a). Rats were forced to run with a 5% incline [5, 6]. Healthy rats were forced to run 30 km (20 m/min for 50 min, 5 days a week) over 6 weeks (Fig. 1b), and we examined the completion rate every week. Rats were observed during the running, and rats which stopped running in the grid over one minute despite electric stimulation were defined as drop out.

### 15 km running of rats after MIA injection

**Fig. 2:**
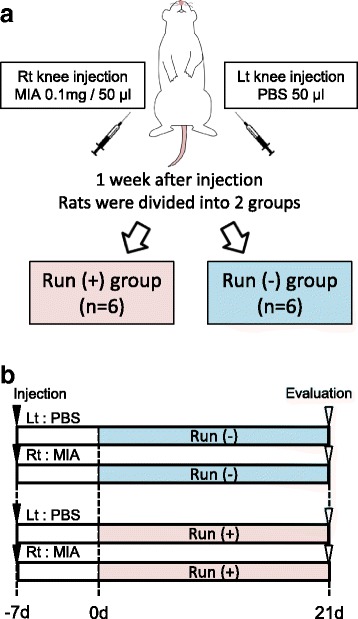
Outline of the study. **a** Schema to show the grouping of knee injection and running. **b** Protocol for injection of mono-iodoacetate (MIA), running, and evaluation

MIA (Sigma-Aldrich, St. Louis, MO, USA) was dissolved in phosphate-buffered saline (PBS). 0.1 mg MIA in 50 μl PBS was injected once into the right knee and PBS was injected into the left knee with a 27-gauge needle in a 1.0 ml syringe through the lateral infrapatellar area toward the inter condylar space of the femur in a deep knee flexed position (Fig. 2a). Seven days after the injection, rats were forced to run 15 km (20 m/min for 50 min, 5 days a week) over 21 days and were then evaluated. Rats were also kept without running for 21 days and evaluated (Fig. 2b).

### Evaluation of synovitis by MIA and 15 km running

First, 7 days after PBS injection, rats were forced to run 15 km over 21 days. Synovium was evaluated in rats before and after 15 km running (Fig. 5a). Second, 7 days after MIA injection, rats were forced to run 15 km over 21 days. Synovium was evaluated in rats before, after 15 km running, and after 21 days without running (Fig. 5d).

### Macroscopic Observation

Femoral and tibial condyles were dissected separately without damaging the cartilage surface, and then stained with India ink to assess cartilage degeneration. Quantification of macroscopic images was evaluated by the area of macroscopic cartilage degeneration. Macroscopic pictures were taken using a ZEISS Stimi 2000C microscope (Carl Zeiss, Oberkochen, Germany) on a dedicated medical photography platform. The degeneration area of the medial tibial plateau was measured using AxioVision Rel software version 4.8 (Carl Zeiss, Oberkochen, Germany). Macroscopic assessment was performed separately by two examiners and the average values were recorded.

### Histology

The distal femur, proximal tibia and whole knee joints were fixed in 4% paraformaldehyde for 7 days, decalcified in 20% EDTA solution for 21 days, and then embedded in paraffin wax. The specimens were sectioned in the sagittal plane at 5 μm and stained with safranin-o/fast green. Each section was evaluated with the Osteoarthritis Research Society International histological grading system (OARSI score: 0 to 24) for articular cartilage degeneration [17]. For evaluation of synovium, 7 days after MIA injections, before and after 15 km of running, whole knee joints were sectioned in a sagittal plane and stained with hematoxylin-eosin (HE). Each section was evaluated with Krenn’s synovitis grading system (Synovitis score: 0 to 9) for infrapatellar fat pad (IFP) synovitis [18]. Histologic sections were visualized using an Olympus BX53 microscope (Olympus, Tokyo, Japan). Microscopic assessment was performed separately by two examiners and the average values were recorded.

### Statistical methods

Statcel 3 (OMS publishing Inc. Saitama, Japan) was used for statistical analyses. Data was assessed by Kruskal-Wallis test and Steel-Dwass post-hoc pairwise test. P-values <0.05 were considered to be statistically significant.

### Systematic review

We searched articles published in January, 1980 to May, 2016 by PubMed. The key words were “running”, “osteoarthritis”, and “rats”. 26 articles were hit and 7 articles were finally selected as “reports on the effects of running exercise on articular cartilage in Wistar rats”.

## Results

### Fifty percent of rats completed the 30 km running regimen

First, rats were forced to run for 30 km over 6 weeks without MIA injections (Fig. 1a, b). Even though all rats run up to 15 km, the number of rats that could not complete the running increased after 20 km. 50% of rats completed 30 km of running (Fig. 1c).

### Strenuous running exacerbated tibial cartilage in the MIA-treated knee

**Fig. 3:**
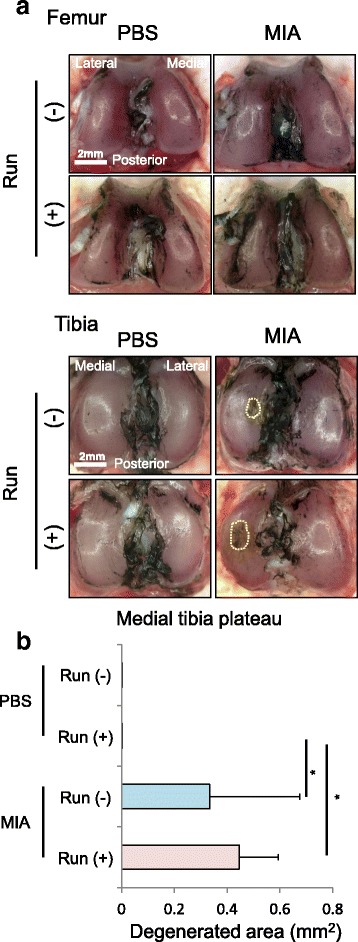
Macroscopic observation for articular cartilage. **a** Macroscopic images of the femoral and tibial articular cartilages stained with India ink. Cartilage erosion is surrounded by a yellow dotted line. **b** Quantification of degenerated area of the tibial cartilage (n =4, * p < 0.05 by Steel-Dwass test)

Secondly, we examined the influences of forced running and MIA injection on cartilage (Fig. 2a, b). All rats completed 15 km of running even after MIA injection. Macroscopically, 15 km of strenuous running affected neither the femoral nor tibial articular cartilage (Fig. 3a). Intra-articuar injection of 0.1 mg MIA did not affect the femoral articular cartilage surface, but induced erosion at the tibial cartilage irrespective of the forced 15 km of running (Fig. 3b).

**Fig. 4:**
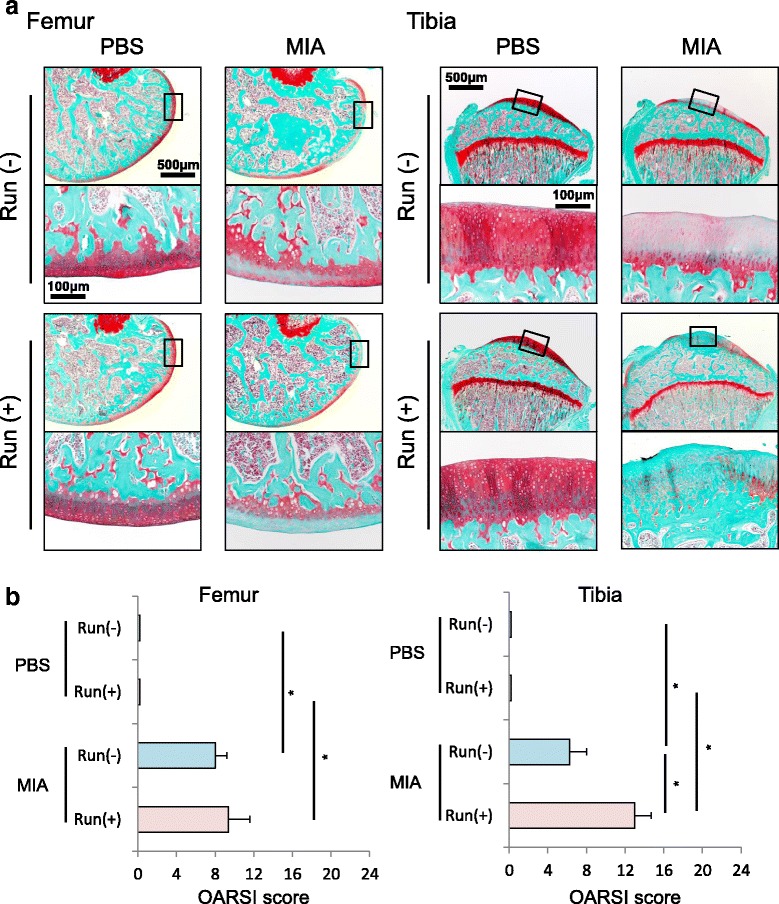
Histological observation for articular cartilage. **a** Femoral and tibial sections stained with safranin-o in the sagittal plane. **b** OARSI scores for femoral and tibial cartilage (n =4, * *p* < 0.05 by Steel-Dwass test)

Histologically, no changes in articular cartilage was found in rat knee joints forced to run 15 km. MIA injection did clearly induced a loss of sulphated-glycosaminoglycans (sGAG) in both femoral and tibial cartilages (Fig. 4a). On the tibial side, cartilage degeneration induced by MIA was observed around the apex part of the tibia, which was not covered with the meniscus. Though the forced running did not affect the femoral cartilage, the forced running further exacerbated the sGAG loss of the cartilage affected by MIA in the tibial cartilage (Fig. 4b). OARSI score for histology was significantly higher in the MIA-treated groups than in the MIA untreated groups both in the femoral and tibial cartilages (Fig. 4b). Interestingly, in the tibial cartilage, OARSI score was significantly higher in the running group than in the non-running group in the MIA-treated condition. This difference was attributed not to stage (area volume) but to grade (tissue reaction); in the MIA treated non-running group, the tibial grade was 2 (cartilage matrix depletion into upper 1/3) or 3 (cartilage matrix depletion into lower 2/3), while in the MIA treated running group, the tibial grades had risen to 3 or 4 (cartilage matrix loss).

### Synovitis caused by MIA was not exacerbated by running

**Fig. 5:**
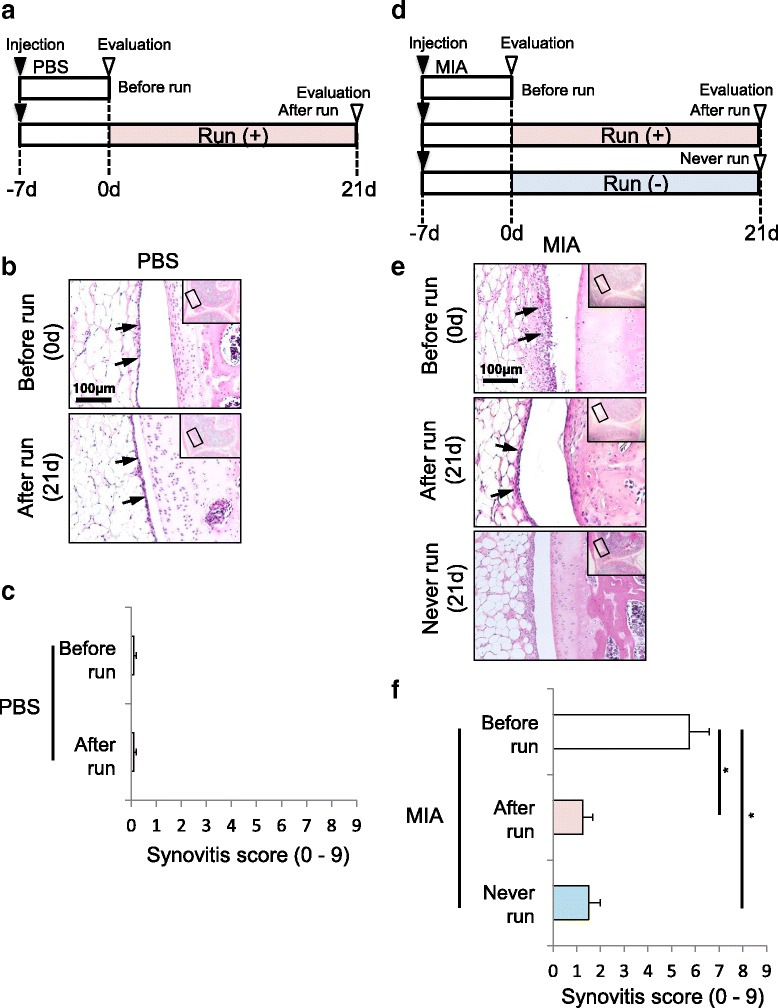
Analyses for synovitis of the knee. **a** Protocol for running without MIA injection. **b** Synovial sections stained with HE. Whole knee joints were sectioned in the sagittal plane for synovium of infrapatellar fat pad. **c** Quantification of synovitis evaluated by Krenn’s synovitis scoring system (*n* = 4). **d** Protocol for running with MIA injection. **e** Synovial sections stained with HE. Synovial cell layer is indicated by arrows. **f** Quantification of synovitis evaluated by Krenn’s synovitis scoring system (*n* = 4, * *p* < 0.05 by Steel-Dwass test)

Thirdly, we examined the influences of forced running and MIA injection on synovium. In the PBS injected knee (Fig. 5a), thickness of the synovial cell layer at the infrapatellar fat pad did not increase after 7 days, the synovial thickness appeared to be unchanged after 15 km of running (Fig. 5b) and synovitis scores were similar between rats not forced to run and rats subjected to forced running (Fig. 5c). In MIA treated knees (Fig. 5d), even though the thickness of the synovial cell layer at the infrapatellar fat pad increased 7 days after MIA injection, the synovial thickness decreased irrespective of running at 21 days (Fig. 5e). The synovitis score in rats injected with MIA at 21 days with or without running was significantly lower than that in rats injected with MIA at 0 days (Fig. 5f). There was no difference between synovitis scores with running and without running in MIA treated knees.

## Discussion

In this study, we revealed that only 50% of the rats completed 30 km of running. 0.1 mg MIA induced cartilage matrix depletion at the tibial cartilage and 15 km of running further exacerbated, even though 15 km running did not affect the cartilage in the PBS injected knee. Synovitis caused by MIA improved with time whether or not rats were forced to run.

As an OA model in rats, 15 km of running was insufficient and 30 km of running induced OA but only 50% of the rats completed the regimen. This is the first report to reveal dropout rate in 30 km of running. To induce OA in rats, 30 km of running in 6 weeks is currently popular but some modifications are required to reduce the dropout rate. Beckett et al. reported that over 30 km running distances were required to induce OA progression in 3 weeks [19]. Even though the drop off rate was not mentioned in this paper, it might increase because 30 km of running in 3 weeks seems more stressful.

The influence of strenuous running on OA remains a matter of controversy. There are 8 reports (including this one) describing the influence of strenuous running on articular cartilage in Wistar rats (Table 1) [5, 19–24]. Thirty km of strenuous running was harmful without other OA inductions in all four papers [5, 19, 20, 24]. Fifteen km of strenuous running with other OA inductions was also harmful in two papers in addition to this one; two other papers reported running as beneficial. Cifuentes et al. reported that 15 km of running over 8 weeks was beneficial in the knee injected with 1.2 mg MIA [23]. The difference between the study by Cifuentes et al. and ours was the time from MIA injection to the start of strenuous running. Cifuentes et al. started strenuous running just after MIA injection, while we did 1 week after injection at which time synovitis was already induced.

According to another study of ours, 0.1 mg MIA induced punctate depressions on the surface of cartilage and cartilage erosion proceeded with time in Wistar rats [10]. In our current results, additional running advanced cartilage degeneration induced by 0.1 mg MIA in only the tibial cartilage, not in the femoral cartilage. In the tibial side, cartilage degeneration induced by MIA was observed around the apex part of the tibia, which was not covered with the meniscus. Therefore, the apex part of the tibia cartilage was possibly sensitive to the mechanical stress induced by strenuous running. In the femoral side, while cartilage degeneration by MIA was observed in the posterior part, mechanical stress would affect an extensive area, not a focal area, of the femoral condyle cartilage. This might reduce the influence of mechanical stress in the femoral cartilage.

0.1 mg MIA induced cartilage degeneration and 15 km of running further exacerbated this at the tibial cartilage. However, this was due to increased proteoglycan loss, which might be reversible and is not necessarily attributable to OA. To make an irreversible OA model, the amount of MIA and the distance of strenuous running must be adjusted in more detail.

Synovitis caused by MIA was observed 7 days after injection; however, synovitis was reduced after 3 weeks with or without 15 km of strenuous running. Additionally, only 15 km of running did not induce synovitis. This means that the strenuous running did not exacerbate and influence synovitis in this model. However, we cannot exclude qualitative or quantitative effects of running that might not be detected by Krenn's score (e.g. changes in cytokine expression, macrophage subtypes, etc.)

To examine the effect of MIA, we tested both treatments (0.1 mg MIA and PBS alone) in the same animal, with each knee receiving a different treatment, because we wished to exclude inter-animal variability. This matched analyses enabled to examine the effect of MIA in more strict manner. However, the use of the contralateral knee as a control is potentially problematic. Gait abnormalities could lead to abnormal stresses on the saline-injected contralateral knee which therefore might not be normal.

In our study, macroscopic and microscopic assessments were performed separately by two examiners. One of the examiners was the author, therefore, evaluation was not completely blinded. The potential for unintentional bias must be taken into consideration.

Mechanical stress is an important factor that regulates cartilage metabolisms [25]. Appropriate mechanical stress maintains or increases the amount of sGAG, while excessive mechanical stress decreases it. MIA induces inflammation, inhibits glycolytic system, reduces viability of chondrocytes, and results in degeneration of the cartilage [9]. In this low dosage of the MIA-induced model, forced running further progressed cartilage degeneration in an inflammatory condition. Both mechanical stress and inflammation are the most important factors affecting OA progression, and even though some modifications are required, this is a good model for mimicking OA in rats.

## Conclusion

0.1 mg MIA induced erosion at the tibial cartilage and an additional 15 km of running for 3 weeks further exacerbated the erosion without dropout in rats.

## Abbreviations

MIA: Mono-iodoacetate
OA: Osteoarthritis
OARSI: Osteoarthritis Research Society International
PBS: Phosphate buffered saline
sGAG: Sulphated-glycosaminoglycans
